# PP07 Developing A Health Technology Assessment Glossary: A Design Thinking Approach To Enhance Health Technology Assessment – Judiciary Communication

**DOI:** 10.1017/S0266462325102274

**Published:** 2025-12-29

**Authors:** Cecilia Menezes Farinasso, Rafael Leite Pacheco, Ana Luiza Cabrera Martimbianco, Camila Monteiro Cruz, Carolina de Oliveira Cruz Latorraca, Isabela Porto de Toledo, Patrícia do Carmo Silva Parreira, Roberta Borges Silva, Verônica Colpani, Rachel Riera

## Abstract

**Introduction:**

Brazil’s health-related litigation has highlighted the need for effective communication between health technology assessment (HTA) and legal professionals. The Hospital Sírio-Libanês Center of HTA (NATS-HSL) in Brazil collaborates with the National Council of Justice to provide capacity building initiatives. This study aimed to present the development of an HTA glossary tailored to judges and legal staff.

**Methods:**

The project followed a Design Thinking methodology with five stages: empathize, define, ideate, prototype, and test. Initially, NATS-HSL conducted an electronic survey of a sample of Brazilian judges to identify preferred terminology and glossary format (empathize stage). During the define and ideate stages, challenges and gaps in interpreting scientific findings were analyzed. The prototype glossary will be produced in print, digital, and PDF formats. These versions will be distributed to legal professionals for review, and their feedback will guide subsequent revisions before implementation.

Sources of Support: Program to Support the Institutional Development of the Unified Health System (PROADI-SUS), Ministry of Health, NUP: 25000.175715/2023-41.

**Results:**

The survey was answered by 144 participants through to November 2024. Among them, 99 (69%) selected an alphabetical glossary format. The most favored formats were online PDF (n=63; 44%) and website (n=50; 35%). Most respondents stated the importance of brevity, the inclusion of examples and “learn more” links (n=97; 67%), the use of informal language (n=93; 65%), and comprehensive terminology (n=90; 63%). The terms most frequently suggested were related to medicines, cost-effectiveness analysis, technical terms, and economic evaluations. The pilot glossary currently contains 210 terms.

**Conclusions:**

The next stages of the glossary development process are prototype distribution, feedback gathering, re-evaluation, and implementation of the final version. This knowledge translation initiative aims to enhance the capacity of legal decision-makers to interpret HTA reports. A better-informed judicial decision may contribute to the sustainability of the healthcare system.

